# Resistance of Lung Cancer Cells Grown as Multicellular Tumour Spheroids to Zinc Sulfophthalocyanine Photosensitization

**DOI:** 10.3390/ijms160510185

**Published:** 2015-05-05

**Authors:** Sello Lebohang Manoto, Nicolette Nadene Houreld, Heidi Abrahamse

**Affiliations:** Laser Research Centre, Faculty of Health Sciences, University of Johannesburg, Doornfontein 2028, South Africa; E-Mails: jakonda@webmail.co.za (S.L.M.); nhoureld@uj.ac.za (N.N.H.)

**Keywords:** apoptosis, resistance, cytotoxicity, spheroids

## Abstract

Photodynamic therapy (PDT) is phototherapeutic modality used in the treatment of neoplastic and non-neoplastic diseases. The photochemical interaction of light, photosensitizer (PS) and molecular oxygen produces singlet oxygen which induces cell death. Zinc sulfophthalocyanine (ZnPcS_mix_) has been shown to be effective in A549 monolayers, multicellular tumor spheroids (MCTSs) (250 µm) and not on MCTSs with a size of 500 µm. A549 cells used in this study were grown as MCTSs to a size of 500 µm in order to determine their susceptibility to PDT. ZnPcS_mix_ distribution in MCTSs and nuclear morphology was determined using a fluorescent microscope. Changes in cellular responses were evaluated using cell morphology, viability, proliferation, cytotoxicity, cell death analysis and mitochondrial membrane potential. Untreated MCTSs, showed no changes in cellular morphology, proliferation, cytotoxicity and nuclear morphology. Photoactivated ZnPcS_mix_ also showed no changes in cellular morphology and nuclear morphology. However, photoactivated ZnPcS_mix_ resulted in a significant dose dependant decrease in viability and proliferation as well as an increase in cell membrane damage in MCTSs over time. ZnPcS_mix_ photosensitization induces apoptotic cell death in MCTSs with a size of 500 µm and more resistantance when compared to monolayer cells and MCTSs with a size of 250 µm.

## 1. Introduction

Lung cancer is the leading cause of cancer death worldwide in both males and females, with an estimated death of 1.4 million each year [1]. Effective treatment of lung cancer remains a challenging problem to date, with lung cancer accounting for 18.2% of the total cause of cancer death, followed by stomach (9.7%) and liver cancers (9.2%) [2]. Although surgery and multimodal therapy such as radiotherapy, immunotherapy, chemotherapy and targeted therapy might improve the overall survival, an effective treatment modality with minimal complications is needed [3]. Photodynamic therapy (PDT) is a promising treatment modality used in the treatment of cancerous and non-cancerous diseases [4].

PDT involves the photochemical interaction of light, photosensitizer (PS) and oxygen, which results in the generation of ROS leading to damage of organelles within the cell. Photofrin and hematoporphyrin are the most widely studied photosensitizers in experimental and clinical trials [5]. Photofrin has been clinically approved by the Food and Drug Administration (FDA, USA) for the treatment of cancer. The disadvantages of this drug are the high degree of chemical heterogeneity, long lasting cutaneous photosensitivity and poor absorption of tissue penetrating red light [6]. To circumvent the limitations of the first generation photosensitizers, scientists are focusing on research of synthesis and testing of new photosensitizers, and among these are phthalocyanines, which are known as second generation photosensitizers. In this study we investigated the properties of a second generation PS, ZnPcS_mix_ as a potential photosensitizing agent. ZnPcS_mix_ contains (Zn) diamagnetic central ion and has an electron absorption spectra of 673 nm in phosphate buffered saline (PBS).

Most cell based assays make use of monolayer or suspension cultures, and the cellular environment of these cultures does not correspond to that of *in vivo* studies, and the outcome of this has little value in predicting the clinical efficacy [7]. Multicellular tumor spheroids (MCTSs) serve as an important model in cancer research for the evaluation of therapeutic interventions since they mimic different aspects of the human tumour tissue environment. MCTSs are three-dimensional tumour cell aggregates that represent cell models intermediate in complexity between two-dimensional monolayer cultures *in vitro* and transplanted tumors *in vivo* [8]. The MCTS culture system is a classical approach to maintain the phenotype of *in vivo* human tumour cells.

The first use of MCTS was in the early 1970s by Sutherland and colleagues for experimental radiation therapy on animal cell lines. Following that, MCTS were later used in PDT, hyperthermia and chemotherapy [9]. Treatment efficacy is predominantly expected to decrease in the 3D pathophysiological environment and this factor makes MCTS a better tool for testing new drugs. The cell to cell or cell to matrix interaction found in spheroids have an effect on hormones and growth factors, as well as the penetration and action of drugs [7]. There is a decrease in oxygen, nutrients, proliferation and metabolites from the outer part to the inner part of the MCTS. MCTS have a necrotic core and the size of this necrotic core varies according to spheroid diameter, cell type, oxygen gradient, pH and nutrients [10].

Several studies show that genes associated with cell survival, resistance, differentiation and proliferation are differentially expressed in MCTSs as compared to monolayer cell cultures [11,12]. The expression of these genes resemble that of the *in vivo* situation, and thus is important for the testing of new drugs using MCTSs. In our previous study, we demonstrated that lung cancer cells grown as MCTSs to a size of 250 µm were more susceptible to PDT as compared to cells cultured as a monolayer. The aim of the present study was to evaluate the cytotoxic effects of PDT in MCTSs with a size of 500 µm using the same parameters as those that were used in our previous study [3]. We also determined whether apoptosis or necrosis was the responsible mode of cell death.

## 2. Results and Discussion

### 2.1. Results

#### 2.1.1. Zinc Sulfophthalocyanine (ZnPcS_mix_) Distribution in Multicellular Tumor Spheroids (MCTSs)

**Figure 1:**
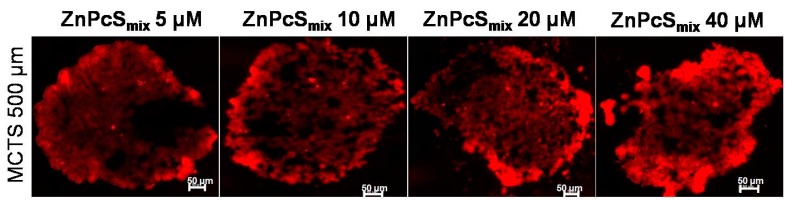
The fluorescence pattern of Zinc Sulfophthalocyanine (ZnPcS_mix_) in multicellular tumour spheroids (MCTSs) with 500 µm. MCTSs treated with a concentration of 40 µm exhibited more red fluorescence intensity as compared to other concentrations. Scale bar denotes 50 µm.

The distribution of ZnPcS_mix_ in MCTS with a size of 500 µm, 24 h post incubation is illustrated in Figure 1. MCTSs treated with increasing concentrations of ZnPcS_mix_ (5, 10, 20 and 40 µM) showed an increase in the red fluorescence intensity as the photosensitizer (PS) concentration increased. There was a strong red fluorescence pattern in the outer rim of the MCTSs. ZnPcS_mix_ penetrates deeper into the inner core of MCTSs, since the red fluorescence can be visualized in the inner core of the MCTSs. MCTS incubated with the highest concentration of ZnPcS_mix_ (40 µM) showed the strongest red fluorescence intensity.

#### 2.1.2. Cellular Morphology

**Figure 2:**
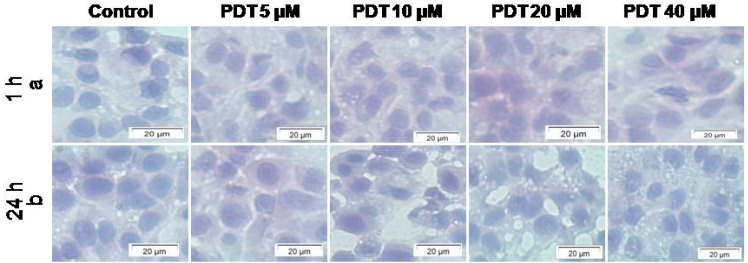
Cryosections of A549 multicellular tumour spheroids (MCTSs) with a size of 500 µm stained with haematoxylin and eosin (H&E) stain, 1 h (**a**) and 24 h (**b**) post irradiation. Control MCTSs were exposed to neither light nor PS while other groups were treated with increasing concentrations of ZnPcS_mix_ (5, 10, 20 and 40 µM) and 5 J/cm^2^. Untreated MCTSs appeared as large cells with prominent cytoplasm and well defined borders 1 or 24 h post incubation. There were no morphological differences in the control MCTSs and those treated with ZnPcS_mix_ (5, 10, 20 and 40 µM) and 5 J/cm^2^. MCTSs appeared as large cells with prominent cytoplasm and well defined borders.

The changes in cellular morphology were assessed using light microscopy. Cryosections of untreated control MCTSs with a size of 500 µm exposed to neither light nor PS and stained with haematoxylin and eosin (H&E) appeared normal with abundant cytoplasm and well defined borders, 1 or 24 h post incubation (Figure 2). No morphological differences were seen between untreated MCTSs, and MCTSs treated with increasing concentrations of ZnPcS_mix_ (5, 10, 20 and 40 µM) and laser irradiation at 5 J/cm^2^, 1 or 24 h post incubation.

#### 2.1.3. Cellular Viability

Cellular viability in A549 cells cultured as MCTSs was evaluated using Trypan blue (Table 1). MCTSs (500 µm) incubated with increasing concentrations of ZnPcS_mix_ alone (5, 10, 20 and 40 µM) showed no significant changes in cellular viability as compared to untreated control cells exposed to neither light nor PS 1 or 24 h post incubation. Photoactivated ZnPcS_mix_ at concentrations of 5 and 10 µM showed no significant changes in cellular viability when compared to untreated control cells 1 h post PDT. However, photoactivated ZnPcS_mix_ at concentrations of 20 and 40 µM resulted in a significant decrease in cellular viability as compared to untreated control cells 1 h post PDT (*p* < 0.001). Photoactivated ZnPcS_mix_ at a concentration of 5 µM showed no changes in cellular viability as compared to the untreated control cells, while there was significantly reduced cellular viability in cells with photoactivated ZnPcS_mix_ at concentrations of 10, 20 and 40 µM, 24 h post PDT (*p* < 0.001).

**Table 1:** Dose response of multicellular tumour spheroids (MCTS) (500 µm) treated with ZnPcS_mix_ at various concentrations (0, 5, 10, 20 and 40 μM) and irradiated using 0 or 5 J/cm^2^. Trypan blue was used to determine percentage viability, while adenosine triphosphate (ATP) was used to measure cell proliferation and luminescence was recorded as relative light units (RLU). Cytotoxicity was determined using lactate dehydrogenase (LDH) assay and absorbance was measured at 490 nm. Statistical differences between untreated controls and the experimental groups are shown as *** *p* < 0.001. Experiments were repeated four times (*n* = 4). ^a^ Mean; ^b^ Standard error.

Cell Function	Fluence	0 µM		5 µM		10 µM		20 µM		40 µM	
		Monolayer	MCTS	Monolayer	MCTS	Monolayer	MCTS	Monolayer	MCTS	Monolayer	MCTS
**Viability**	0 J/cm^2^	88.35 ^a^ ± 3.50 ^b^	85.5 ± 2.22	97.25 ± 0.25	84.00 ± 1.29	96.75 ± 0.48	89.75 ± 2.63	98.5 ± 0.288	87.00 ± 2.55	96.75 ± 1.11	85.00 ± 3.29
	5 J/cm^2^	91.75 ^a^ ± 2.06 ^b^	89.25 ± 2.17	69.75 *** ± 1.65	55.5 *** ± 3.59	45.75 *** ± 3.61	33.5 *** ± 1.55	39.00 *** ± 9.05	25.00 *** ± 2.68	37.25 *** ± 4.385	21.25 *** ± 1.31
**Proliferation**	0 J/cm^2^	279,325 ± 1328.11	23,629 ± 700.93	313,316 ± 5179.75	25,332 ± 2609.47	319,120 ± 11,247.98	28,879 ± 890.52	289,718 ± 14,702.04	27,987 ± 1133.90	277,319 ± 21,847.83	25,691 ± 1478.26
	5 J/cm^2^	285,533 ± 8139.17	21,129 ± 431.37	19,450 *** ± 3414.49	832 *** ± 99.22	175,827 *** ± 1592.56	2796 *** ± 393.48	140,123 *** ± 3883.81	1179 *** ± 90.88	5809 *** ± 364.67	420 *** ± 66.94
**Cytotoxicity**	0 J/cm^2^	0.343 ± 0.02	0.249 ± 1.18	0.339 ± 9.71	0.25 ± 2.43	0.33 ± 0.01	0.26 ± 0.01	0.36 ± 8.31	0.23 ± 3.3	0.37 ± 0.01	0.232 ± 0.11
	5 J/cm^2^	0.288 ± 0.02	0.249 ± 1.18	1.803 *** ± 0.14	0.876 *** ± 0.07	2.146 *** ± 0.17	0.851 *** ± 0.09	1.720 *** ± 0.12	0.882 *** ± 0.07	2.185 *** ± 0.34	0.569 *** ± 0.02

#### 2.1.4. Cellular Proliferation

The CellTiter-Glo^®^ luminescent assay measured the number of proliferating cells in culture. The incubation of MCTSs (500 µm) with ZnPcS_mix_ alone at increasing concentrations of 5, 10, 20 and 40 µM produced no significant changes in cellular proliferation when compared to untreated control cells exposed to neither light nor PS 1 or 24 h post incubation (Table 1). Photoactivated ZnPcS_mix_ (5, 10, 20 and 40 µM) resulted in a significant dose dependant decrease in cellular proliferation as compared to untreated control cells 1 and 24 h post PDT (*p* < 0.001) except at a concentration of 5 µM after 1 h of PDT (*p* = 0.37).

#### 2.1.5. Cellular Cytotoxicity

The CytoTox96^®^ Assay was used to measure the release of LDH, thereby evaluating cell membrane damage. MCTSs with a size of 500 µm incubated with ZnPcS_mix_ alone at increasing concentrations of 5, 10, 20 and 40 µM resulted in no statistical significance in the amount of LDH released as compared to untreated control cells exposed to neither light nor PS (Table 1). Photoactivated ZnPcS_mix_ at concentrations of 5 and 10 µM produced a significant increase in the amount of LDH released as compared to untreated control cells 1 h post PDT (*p* < 0.001 and *p* < 0.05 respectively). No significant changes were noted in photoactivated ZnPcS_mix_ at concentrations of 20 and 40 µM as compared to untreated control cells after 1 h of PDT. Photoactivated ZnPcS_mix_ at concentrations of 5, 10, 20 and 40 µM resulted in a significant increase in the release of LDH when compared to untreated control cells 24 h post PDT (*p* < 0.01; *p* < 0.001; *p* < 0.001 and *p* < 0.001 respectively).

#### 2.1.6. Cell Death Analysis

**Figure 3:**
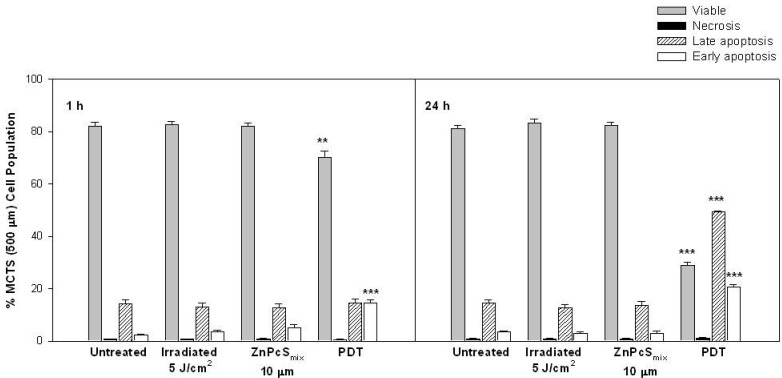
Evaluation of different stages of cell death as determined by flow cytometric analysis in multicellular tumour spheroids (MCTSs) with a size of 500 µm (*n* = 4). Photoactivated ZnPcS_mix_ at 10 µM and 5 J/cm^2^ resulted in a significant decrease in viable cells and an increased percentage population of early apoptotic cells 1 h post PDT. After 24 h, PDT resulted in a further reduction in viable cells and an increased population of late apoptotic cells. Significant differences between untreated controls and the experimental groups are represented in the graph as ** *p* < 0.01 or *** *p* < 0.001.

The Annexin V-FITC apoptosis detection kit was used in conjunction with PI to discriminate between apoptotic and necrotic cells using flow cytometry. MCTSs (500 µm) irradiated using 5 J/cm^2^ showed no significant changes in percentage of cell population as compared to untreated control cells exposed to neither light nor PS 1 or 24 h post incubation (Figure 3). ZnPcS_mix_ alone at 10 µM also showed no significant changes in percentage of cell population when compared to untreated control cells 1 or 24 h post incubation. Photoactivated ZnPcS_mix_ (10 µM) had a significant decrease in the proportion of viable cells 70% (*p* < 0.01) and a significant increase in early apoptotic cells of 14.7%, 1 h post PDT (*p* < 0.001). No statistical significance was seen 1 h post incubation when the late apoptotic and necrotic proportion of the untreated control cells was compared to the late apoptotic and necrotic proportion in the PDT treated cells. Photoactivated ZnPcS_mix_ (10 µM) had a significant decrease in the proportion of viable cells 28.8% (*p* < 0.001) and a significant increase in early apoptotic 20.7% (*p* < 0.001) and late apoptotic 49.3% (*p* < 0.001) cells 24 h post PDT. MCTSs (500 µm) 24 h post PDT showed a high proportion of late apoptotic cells when compared to 1 h (*p* < 0.001).

#### 2.1.7. Mitochondrial Membrane Potential

**Figure 4:**
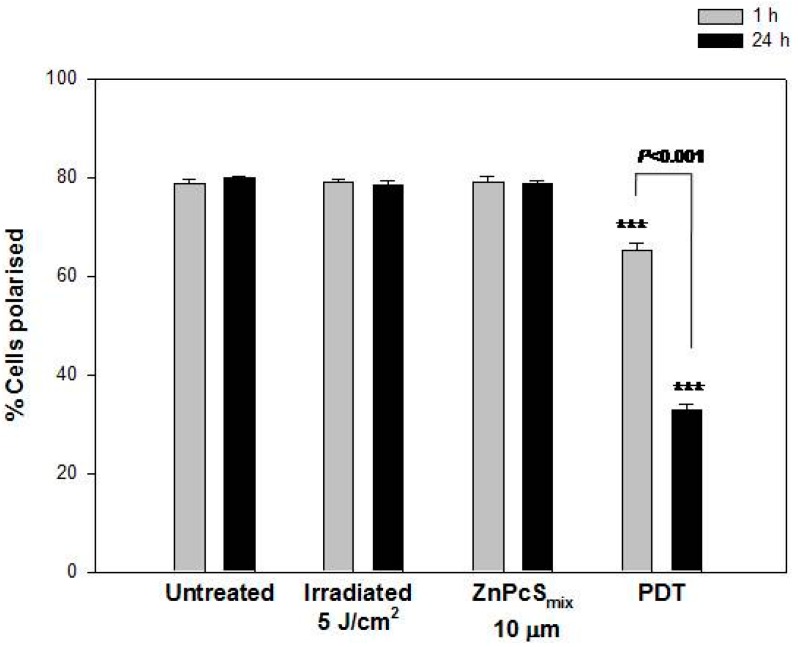
Mitochondrial membrane potential (MMP) was evaluated using JC-1 dye by flow cytometry (*n* = 4). Untreated multicellular tumour spheroids (MCTSs) showed an increased percentage of polarized cells 1 and 24 h post PDT. Irradiated MCTS and those that received ZnPcS_mix_ (10 µM) in the absence of light also showed an increased percentage of polarized cells. There was a reduction in the percentage of polarized cells in 1 or 24 h post PDT (*p* < 0.001). Significant differences between untreated controls and the experimental groups are represented in the graph as *** *p* < 0.001.

JC-1 dye was used to evaluate the status of the mitochondrial membrane potential (MMP) (Figure 4). The MMP of normal, healthy cells is polarized and JC-1 is rapidly taken up by cell mitochondria. During apoptosis or other cellular processes there is an altered mitochondrial function resulting in depolarized MMP. Cells cultured as MTCS showed an in increased percentage of polarized cells, 1 or 24 h post incubation. The irradiation of MCTSs using 5 J/cm^2^ showed no significant changes in the percentage of polarized cells as compared to untreated control cells exposed to neither light nor PS 1 or 24 h post incubation. ZnPcS_mix_ alone at 10 µM in MCTSs also showed no significant changes in the percentage of polarized cells when compared to their respective untreated control cells 1 or 24 h post incubation. Photoactivated ZnPcS_mix_ (10 µM) resulted in a decrease in the percentage of polarized cells as compared to their respective untreated control cells 1 or 24 h post PDT (*p* < 0.001). Photoactivated ZnPcS_mix_ (10 µM) 24 h post PDT showed less polarized cells when compared 1 h post PDT (*p* < 0.001). The decrease in the percentage of polarized cells after PDT may be associated with apoptosis seen by Annexin V-FITC.

#### 2.1.8. Nuclear Morphology

**Figure 5:**
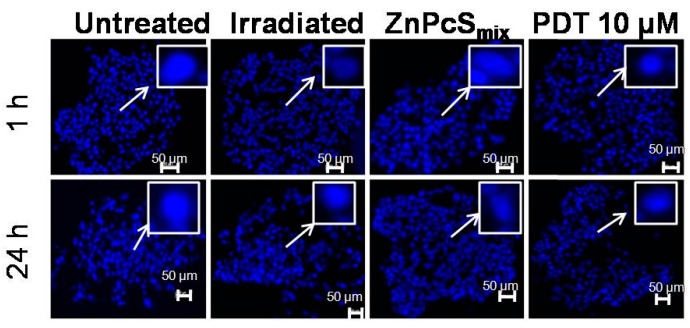
Fluorescence micrograph of multicellular tumour spheroids (MCTSs) with a size of 500 µm stained with Hoechst 33342, 1 or 24 h post incubation. Scale bar denotes 50 µm in MCTSs. The arrows show enlarged nuclei.

Hoechst 33342 was used to evaluate apoptosis since it allows the visualization of nuclear condensation and fragmentation seen in apoptosis as well as the internal organization of MCTSs (Figure 5). The nuclei of MCTSs (500 µm) irradiated with 5 J/cm^2^ alone and incubated with 10 µM of ZnPcS_mix_ alone appeared similar to that of untreated MCTSs, 1 or 24 h post PDT. There was no loss of cohesion in MCTSs with a size of 500 µm 1 or 24 h post PDT. No signs of apoptosis were visible 1 or 24 h post PDT.

### 2.2. Discussion

In this study, we evaluated the effectiveness of a ZnPcS_mix_ in treating lung cancer cells grown as MCTSs. Experiments on monolayer cell culture are routinely used for the testing of anti-tumour agents. However, this monolayer cell culture systems represents an artificial tumour environment and the results cannot be directly transferred to an *in vivo* situation. Three dimensional cultures such as in MCTSs can better mimic the *in vivo* behavior of cells and are increasingly recognized as valuable advanced tools for the testing of therapeutic interventions [7]. Many treatment modalities are expected to have reduced efficacy in three-dimensional pathophysiological environments and the use of MCTS is seen as a tool for eliminating the testing of ineffective drugs in animals [9]. Comparison of different cell culture models might lead to a better understanding of the mechanisms involved in the cellular toxicity of ZnPcS_mix_ and what to expect *in vivo* [4].

The fluorescence distribution of ZnPcS_mix_ in MCTS is non-uniform with increased fluorescence intensity in the outer rim of the MCTSs and reduced fluorescence intensity in the inner core. A reduced fluorescence pattern inside HT29 human adenocarcinoma spheroids using Foscan^®^ as a PS was also demonstrated [13]. The fluorescence intensity of ZnPcS_mix_ in MCTSs increases with an increase in PS concentration. This result suggests that ZnPcS_mix_ penetrates deeper into the MCTS with an increased concentration of ZnPcS_mix_. MCTS size has been shown to have an influence in the penetration of the drug and in this study, ZnPcS_mix_ was able to penetrate MCTSs of large size.

Untreated MCTSs maintained cellular morphology, viability and proliferation, and showed little cell membrane damage. This has been confirmed in other studies that have also reported no changes in cell morphology, increased cell viability and increased cell proliferation in untreated cells [14]. Untreated control MCTSs analyzed using Annexin V-FITC showed increased viability and a decreased percentage of apoptotic cells while JC-1 showed an increased percentage of polarized cells. These results concur with those of [15] where untreated controls showed an increased population of viable cells and a decreased population of early and late apoptosis and necrotic cells, while JC-1 showed an increased percentage of polarized cells.

Irradiated MCTSs using 5 J/cm^2^ showed a similar trend as untreated control cells; there were no changes in cellular morphology, with no changes in cellular viability and proliferation and no cell membrane damage and nuclear changes. Annexin V-FITC and JC-1 revealed the same pattern as untreated cells. A study conducted by [16] showed that the irradiation of oesophageal cells using 20 J/cm^2^ did not alter cell morphology, viability and proliferation. The use of an irradiation dosage that does not have a stimulatory effect on cancer cells is required in PDT as this can promote the proliferation of cells. The 5 J/cm^2^ used in this study does not stimulate the proliferation of lung cancer cells grown as MCTSs and can fully activate the PS. Similarly to that of the untreated control, irradiated MCTSs showed a decrease in cellular viability, proliferation and polarized cells in comparison. For MCTS treated with ZnPcS_mix_ alone a similar trend as untreated and irradiated cells was observed. A similar study conducted by [17] showed that there was no significant difference in cellular viability in MCF-7 cells as a result of the addition of ZnPcS_mix_ in the absence of laser irradiation. The normal cell growth of A549 cells grown as MCTS in the presence of the ZnPcS_mix_ indicates that this PS is a good photosensitizer and is not active in the absence of light.

Photoactivated ZnPcS_mix_ showed changes in cellular morphology, decreased viability and proliferation while there was an increase in cytotoxicity. MCTS cryosections with a size of 500 µm showed no changes in morphology 1 or 24 h post PDT. It was reported that MCTS with a size of 250 µm stained with H&E 1 h post PDT showed no changes in morphology while 24 h post PDT there was shrinkage of the cytoplasm and the nuclei, which are features seen in apoptosis [4]. This result indicates that the size of the MCTS plays a critical role in the outcome of PDT. In addition this shows that MCTSs with a size of 500 µm are more resistant to PDT as compared to MCTSs with a size of 250 µm. The results of MCTSs with a size of 500 µm concurred with those in the literature and showed resistance as compared to monolayer and MCTSs with a size of 250 µm [18].

MCTSs show a gradual decrease in oxygen pressure as you move towards the inner core of the spheroid with larger sizes of MCTSs showing the greatest reduction in oxygen [19]. In this study resistance to PDT seen in MCTSs with a size of 500 µm might have been due to this reduced oxygen found in larger MCTSs. Since PDT is an oxygen dependent therapy, a reduction in oxygen will reduce the clinical efficiency. It is well known that the delivery of oxygen *in vivo* to the treatment site can be a limiting factor in PDT [20]. The outer layer of MCTS consists of well-oxygenated proliferating cells, and has been shown to retain the highest levels of PS, and thus is more sensitive than the innermost layer.

The LDH released in MCTSs 500 µm post PDT showed a decreased release as compared to the monolayer cells and MCTSs with a size of 250 µm [4]. The penetration of ZnPcS_mix_ might have played a role in the decreased release of LDH post PDT in MCTSs with a size of 500 µm, since less ZnPcS_mix_ penetrates MCTSs (500 µm) compared to MCTSs (250 µm). The 3D geometry of cells in MCTSs might have contributed to the reduced release of LDH in MCTS.

There were no signs of apoptosis in MCTSs visualized with Hoechst 33342. Hoechst 33342 revealed loss of cohesion in MCTS (250 µm) while there was no loss of cohesion in MCTS (500 µm). These results suggest that Hoechst 3342 might not be an ideal stain for nuclear morphology in cells grown as MCTS. However, the absence of nuclear condensation and fragmentation might also indicate a different mode of cell death after PDT such as autophagy. Autophagic cell death after PDT was demonstrated in prostate cancer (PC-3) cells [21]. PDT can induce different modes of cell death in cultured cells and this is dependent on the experimental conditions. The mode of cell death after photosensitization can either be apoptosis, necrosis or autophagy [22]. In MCTSs with a size of 500 µm, the prevalent population of cells 1 h post PDT were early apoptotic cells (14.7%) whereas 24 h post PDT the dominant cell population were late apoptotic cells (49.3%) and some populations were early apoptotic (20.7%). These results suggests that the membrane integrity was lost in a time dependant manner. Apoptotic cell death has been shown to be a prominent form of cell death in many cell types after photosensitization [13].

JC-1 dye penetrates the plasma membrane of cells as monomers and the uptake into the mitochondria is driven by the MMP. Normal healthy mitochondria is polarized and JC-1 is rapidly taken up into the mitochondria resulting in the formation of JC-1 aggregates leading to higher levels of red fluorescence emission. Apoptosis or other physiological events can results in depolarized MMP causing JC-1 not to accumulate in the mitochondria and remain in the cytoplasm as monomers and therefore reduced levels of the red fluorescence. The loss of MMP seen in this study after ZnPcS_mix_ photosensitization may be caused by the opening of the mitochondrial transition pore and is thought to induce cell death and inhibit cell growth. The opening of the mitochondrial transition pore causes the release of Ca^2+^, which plays a role in the release of cytochrome c by the mitochondria [23]. The loss of MMP in human hepato cellular carcinoma cells using zinc phthalocyanine localizing in the mitochondria was also demonstrated [24]. Activation of phthalocyanine induces apoptosis when there is an increase in intracellular Ca^2+^ concentrations which is directly dependent on the presence of p53 was also reported [25]. A collapse in MMP is a common feature of apoptosis, and since apoptotic cell death was the prominent form of cell death, it is possible that a collapse in the MMP was involved in the apoptotic cell death observed in this study.

## 3. Experimental Section

### 3.1. MCTS Cell Culture

MCTSs were initiated by culturing 5 × 10^4^ A549 cells in 75 cm^2^ flasks, coated with 1% low melting agarose (Sigma Aldrich, A9414, Gauteng, South Africa). Cell aggregates formed after 3 days and were transferred to 250 mL spinner flasks (Integra Biosciences, 182026, Hudson, NH, USA) containing 150 mL of culture medium. The culture medium contained Rosewell Park Memorial Institute 1640 medium (RPMI, Invitrogen, 21875-034, Johannesburg, South Africa) supplemented with 10% fetal bovine serum (FBS, Gibco, 306.00301, Johannesburg, South Africa), 0.5% penicillin/streptomycin (Gibco, 15140) and 0.5% amphotericin-B antifungal (Gibco, 104813). The flasks were placed on a magnetic spinner plate operating at 75 rpm in an incubator at 37 °C in 5% CO_2_ and 85% humidity. Culture medium was changed 3 times weekly by allowing the spheroids to settle to the bottom of the spinner flask, carefully removing 100 mL of the medium and replacing with fresh medium. The spheroids reached a size of 500 µm in diameter in approximately 14 days and were then used for experiments.

### 3.2. ZnPcS_mix_ Distribution in MCTSs

MCTSs were prepared as before and incubated with ZnPcS_mix_ at a concentration of 5, 10, 20 and 40 µM for 24 h at 37 °C in order to determine the localization pattern of ZnPcS_mix._ MCTSs were washed twice in PBS and then 5 MCTSs were collected for cryosectioning. The 5 spheroids were collected using a pipette, placed on Shandon cryomatrix (Thermo Scientific, 6769006, Johannesburg, South Africa) and sectioned at 10 µm using a croyostat. The fluorescence pattern of ZnPcS_mix_ in the MCTS was visualized using the Carl Zeiss Axio Observer Z1 fluorescent microscope.

### 3.3. Photodynamic Treatment of MCTS

A total of 50 A549 spheroids were collected from spinner flasks and transferred to 3.4 cm^2^ diameter culture dishes. Spheroids were treated with ZnPcS_mix_ at various concentrations (0, 5, 10, 20 and 40 μM) for 24 h, in order to determine optimum toxic concentration. Prior to laser irradiation, spheroids were then washed twice with Hanks Balanced Salt Solution (HBSS, Invitrogen, 10-543F) to remove any unabsorbed ZnPcS_mix_ and fresh media was added. The irradiation of spheroids was conducted using a diode laser emitting at wavelengths of 681.5 nm, supplied by the National Laser Center (NLC) South-Africa. The light beam was delivered from the top of the culture dish and was not in direct contact to the culture dish. Laser irradiation parameters are shown in Table 2 and all laser irradiations were performed in the dark. Spheroids were further incubated at 37 °C in 5% CO_2_ and 85% humidity for 1 or 24 h before biochemical responses were assessed.

**Table 2:** Laser irradiation parameters.

Laser Type	Diode
Wavelength	680 nm
Spot size	9.1 cm^2^
Power density	4.87 mW/cm^2^
Energy density (Fluence)	5 J/cm^2^
Output power	44.2 mW
Output mode	Continuous
Duration of exposure	17 min 7 s

### 3.4. Cell Morphology

Changes in cell morphology were determined using an inverted microscope (Wirsam, Olympus CKX41, Johannesburg, South Africa) fitted with a colour camera using imaging analysis software 1 or 24 h post irradiation. Once digital images were recorded, cells were trypsinized using TrypLE™ Express (Invitrogen, 12605-028) and resuspended to perform further assays. For cryosections, 5 spheroids were collected using a pipette, placed on optimal cutting temperature (OCT) compound and sectioned at 10 µm using a cryostat. The sections were stained with hematoxylin and eosin (H&E) stain and visualized using an inverted microscope.

### 3.5. Cellular Viability

The Countess™ automated cell counter (Invitrogen, Johannesburg, South Africa) was used to determine cell viability using the Trypan blue staining technique. Ten microlitres of the cell suspension was added to 10 μL of the supplied 0.4% Trypan blue (Invitrogen, T10282) in a 500 µL centrifuge tube and gently mixed. Ten microlitres of the cell suspension was loaded on a Countess™ slide and the slide was placed into the automated cell counter which counted viable (unstained) and non-viable (stained) cells. Percentage viability was recorded from 5 squares and an average was used to determine cellular viability.

### 3.6. Cellular Proliferation

The CellTiter-Glo^®^ luminescent assay (Promega, G7571, Johannesburg, South Africa) is a homogeneous method for determining cellular proliferation. This assay quantifies adenosine triphosphate (ATP) present in active cells. Fifty microlitres of reconstituted reagent was added to an equal volume of cell suspension and then mixed on a shaker for 2 min to induce cell lysis. This was then incubated at room temperature, protected from light, for 10 min to stabilize the luminescent signal. Luminescent signal was read using the 1420 Multilabel Counter Victor^3^ (Perkin-Elmer, Johannesburg, South Africa).

### 3.7. Cellular Cytotoxicity

The CytoTox96^®^ Assay (Promega, G4000), which measures lactate dehydrogenase (LDH), a cytosolic enzyme that is released upon cell lysis, was used to determine cell membrane damage. The amount of LDH released into the culture media correlates with the amount of cell death and cell membrane damage. Fifty microliters of the substrate was added to an equal volume of culture medium per well, in a flat-bottomed 96 well plate. This was incubated for 30 min protected from light and then 50 µL of the stop solution was added to each well. Absorbance was read at 490 nm using the 1420 Multilabel Counter Victor^3^ (Perkin-Elmer).

### 3.8. Cell Death Analysis

The FITC (fluorescein isothiocyanate) annexin V apoptosis detection kit (Becton Dickinson, 556570, Johannesburg, South Africa) was used to quantitatively determine the percentage of cells undergoing apoptosis by fluorescence activated cell sorting (FACS). During apoptosis, the membrane phospholipid phosphatidylserine, which is normally found in the internal portion of the cell membrane, becomes translocated to the outer leaflet of the plasma membrane, thereby exposing phosphatidylserine to the external environment. Annexin V is a calcium dependant phospholipid binding protein that has an affinity for phosphatidylserine and is useful in identifying apoptotic cells. Cells were resuspended in 1× binding buffer at a concentration of 1 × 10^6^/mL. One hundred microlitres of the cell suspension was transferred to a 5 mL tube and stained with 5 µL of FITC annexin and 5 µL of propidium iodide (PI). PI is a standard flow cytometric viability probe and by was used to distinguish viable from non-viable cells. Viable cells have an intact membrane and will exclude PI while membranes of dead or damaged cells are permeable to PI. The cells were gently vortexed and incubated for 15 min at room temperature protected from light. After the addition of 400 µL of 1× binding buffer to each tube the samples were analyzed on a FACSAria (Becton Dickinson) flow cytometer.

### 3.9. Mitochondrial Membrane Potential

The status of MMP (Mitochondrial membrane potential) was evaluated using the MitoScreen kit (5,5',6,6'-tetrachloro-1,1',3,3'-tetraethylbenzimidazolcarbocyanine iodide, JC-1). JC-1 dye is a membrane permeable lipophilic cationic fluorochrome that is able to penetrate cells and its fluorescence is a reflection of the MMP. MCTSs were collected as before, resuspended in 1 mL media at a concentration of 1 × 10^6^ cell/mL and centrifuged at 400× *g* for 5 min at room temperature. Following centrifugation, the supernatant was carefully removed and discarded. Then 500 µL of freshly prepared JC-1 working solution was added to each tube and vortexed to disrupt any cell to cell clumping. Cells were incubated for 15 min at 37 °C in a CO_2_ incubator and washed twice by adding 2 mL of 1× assay buffer for the first wash and 1 mL of the same buffer for the second wash. Cells were then centrifuged as before and resuspended in 500 µL of 1× assay buffer. The samples were then analyzed on FACSAria (Becton Dickinson) flow cytometer.

### 3.10. Nuclear Morphology

Hoechst 33342 (trihydrochloride trihydrate), which is a blue fluorescent cell permeable nucleic acid stain, was used as a measure of apoptosis since it allows the visualization of nuclear condensation and fragmentation seen in apoptosis. This dye is highly sensitive to DNA conformation and chromatin state of a cell, thereby detecting gradations of nuclear damage. MCTSs were treated with 10 μM of ZnPcS_mix_ and irradiated with 5 J/cm^2^. Cryosections of MCTS were prepared as before and stained with Hoechst 33342 for 5 min, 1 or 24 h post PDT. The slides were observed using the Carl Zeiss Observer Z1 fluorescent microscope (Carl Zeiss, Johannesburg, South Africa).

### 3.11. Statistics

All experiments were performed four times (*n* = 4). Biochemical assays were performed in duplicate and an average of the results was used. Statistical analysis was performed using SigmaPlot software version 8.0 and the mean, standard deviation, standard error and significant changes were calculated. A student *t*-test and ANOVA repeated measure was performed to determine the statistical difference between the control and experimental groups. Statistical differences between the untreated controls and experimental groups are shown in graphs as ** *p* < 0.01, and *** *p* < 0.001 and dispersion bars represent standard error.

## 4. Conclusions

In conclusion, ZnPcS_mix_ shows no dark toxicity, and is inactive in the absence of light in A549 cells grown as MCTSs. Irradiation alone also has no effect on A549 cells in the absence of ZnPcS_mix_. ZnPcS_mix_ accumulates rapidly in cells and induce cell death upon illumination with laser light. Monolayer cells and MCTSs (250 and 500 µm) differ in their response to PDT with MCTSs (250 µm) being the most susceptible model to PDT. The reduction in susceptibility to PDT in MCTSs with a size of 500 µm might be due to a reduced uptake of ZnPcS_mix_ in MCTSs (500 µm) *vs.* smaller MCTSs with a size of 250 µm. The size of the MCTS plays a critical role in the outcome of PDT as demonstrated in this study, since smaller spheroids are less resistant to PDT compared to larger spheroids (Mehta *et al.*, 2012). Photoactivated ZnPcS_mix_ induces cell death by triggering the production of ROS which causes lysosomal membrane permeabilization, mitochondrial swelling and loss of MMP, which may result in the release of cytochrome c causing apoptotic cell death. The most dominant mode of cell death in MCTSs is apoptotic cell death.
